# Doxorubicin combined with low intensity ultrasound suppresses the growth of oral squamous cell carcinoma in culture and in xenografts

**DOI:** 10.1186/s13046-017-0633-y

**Published:** 2017-11-21

**Authors:** Haixia Fan, Haixia Li, Guanyao Liu, Wei Cong, Hong Zhao, Wenwu Cao, Jinhua Zheng

**Affiliations:** 1 Departmentof Anatomy, Basic Medical Science College, Harbin Medical University, 194 Xuefu Road, Nangang District, Harbin, 150081 China; 2 Department of Oral Medicine, Jining Medical College, Shandong, 272067 China; 3 Department of Forensic Medicine, Basic Medical Science College, Harbin Medical University, Harbin, 150081 China; 4 Department of Oral Pathology, Stomatological Hospital, Harbin Medical University, Harbin, 150001 China; 5 Condensed Matter Science and Technology Institute, and Department of Physics, Harbin Institute of Technology, Harbin, 150080 China; 6 Materials Research Institute, The Pennsylvania State University, University Park, Pennsylvania, 16802 USA

**Keywords:** Oral squamous cancer cells, Doxorubicin, Ultrasound, MMP-2/9, Hedgehog signaling pathway

## Abstract

**Background:**

Oral squamous cell carcinoma (OSCC) invades surrounding tissues by upregulating matrix metalloproteinases (MMPs) -2 and −9, which causes over-expression of the Hedgehog signaling proteins Shh and Gli-1 and degradation of the extracellular matrix, thereby creating a “highway” for tumor invasion. We explored the potential of low intensity ultrasound (LIUS) and doxorubicin (DOX) to inhibit the formation of this “highway”.

**Methods:**

MTT assays were used to examine OSCC cell viability after exposure to LIUS and DOX. The cell morphological changes and ultrastructure were detected by scanning electron microscopy and transmission electron microscopy. Endogenous autophagy-associated proteins were analyzed by immunofluorescent staining and western blotting. Cell migration and invasion abilities were evaluated by Transwell assays. Collagen fiber changes were evaluated by Masson’s trichrome staining. Invasion-associated proteins were analyzed by immunohistochemistry and western blotting.

**Results:**

LIUS of 1 W/cm^2^ increased the in vitro DOX uptake into OSCC by nearly 3-fold in three different cell lines and induced transient autophagic vacuoles on the cell surface. The combination of LIUS and 0.2 μg/ml DOX inhibited tumor cell viability and invasion, promoted tumor stromal collagen deposition, and prolonged the survival of mice. This combination also down-regulated MMP-2, MMP-9, Shh and Gli-1 in tumor xenografts. Collagen fiber expression was negatively correlated with the expression of these proteins in human OSCC samples.

**Conclusions:**

Our findings suggest that effective low dosages of DOX in combination with LIUS can inhibit cell proliferation, migration and invasion, which might be through MMP-2/9 production mediated by the Hedgehog signaling pathway.

**Electronic supplementary material:**

The online version of this article (10.1186/s13046-017-0633-y) contains supplementary material, which is available to authorized users.

## Background

Despite rapid progress in chemotherapies, radiotherapies, and targeted gene therapies that are used in conjunction with the mainstay treatment of surgery [1–4], the prognosis of oral squamous cell carcinoma (OSCC) is still poor. This is due to the extremely aggressive and metastatic nature of oral cancers. Tumor invasion and metastasis are dynamic and complicated processes that involve the disruption of the basement membrane (BM) by malignant cells, the degradation of the extracellular matrix (ECM) and the invasion of blood and lymphatic vessels [5]. Proteolysis of the BM and ECM by metalloproteinases (MMPs) is essential for local invasion [6]. Additionally, the Hedgehog (Hh) signaling pathway regulates tumor invasion and metastasis, with its mediators Shh and Gli-1 over-expressed in OSCC [7]. Therefore, the inhibition of collagen fiber degradation in the ECM and BM by MMPs and the modulation of upstream signaling molecules in the Hh pathway may be keys to controlling clinical tumor invasion and migration in OSCC.

Doxorubicin (DOX), which can obviously inhibit the synthesis of DNA and RNA, is one of the most effective anti-cancer drugs in several types of cancers [8, 9]. In combination with LIUS, DOX inhibits the activation of the PI3K/Akt pathway in rat brain glioma [9]. As an upstream signaling activator of PI3K/Akt, the Hh signaling pathway plays an important role in tumor cell proliferation, cell migration and metastasis [10, 11]. Previous research suggests that the Hh signaling pathway is activated in oral cancer [12, 13]. Therefore, DOX may have an advantage over conventional OSCC treatments in that it targets Hh signaling. However, the limitations of DOX have become increasingly evident: DOX has been shown to be associated with increased incidence of cardiomyopathy, and drug tolerance limits its usage in cancer treatment [14, 15]. Moreover, the drug is almost undetectable in tumors and their surrounding areas after intravenous administration of DOX to mice, which indicates that DOX has a very low permeability and diffusion potential [16]. Therefore, an important research goal would be to find a non-invasive approach to improve local drug permeability in OSCC cells.

Extensive investigation of the biological effects of ultrasound on tissues suggests that the use of ultrasound in combination with chemotherapeutic agents or sonosensitizers may provide an important new therapeutic field. Ultrasound provides a non-invasive and effective approach for the delivery of drugs. Some reports indicate that heat generated by ultrasound can increase the sensitivity to chemotherapy, which has been applied in thermal-chemical treatment [17, 18]. It is worth noting, however, that the thermal effects of ultrasonic wave propagation in liquids also increase blood circulation in local tissues, which can promote tumor cell extravasation into the blood vessels to produce metastasis [19].

Initially, autophagy was thought to be a survival mechanism in stress responses such as nutrient deprivation and hypoxia [20]. Autophagy is necessary to maintain internal environmental stability. Studies have shown that autophagy is a priority response to apoptosis after ultrasound treatment, suggesting that it may be cytoprotective in the setting of ultrasound treatment [21]. In addition, inhibition of autophagy can increase the sensitivity of genetic, chemo-, or radio-therapy treatments, which can help to eradicate tumor cells [21, 22].

In our previous study, we demonstrated that LIUS combined with *Scutellarin* or 5-FU can destroy tongue cancer cells and hepatocellular carcinoma cells, with no significant effects on normal cells [23, 24]. The major potential mechanism by which the cell membrane permeability is increased is through mechanical force and cavitation, thereby promoting drug uptake by cancer cells. Furthermore, this treatment method does not easily generate heat, limiting the effect on pathological sites and minimizing the damage to the surrounding normal tissues, which increases its clinical research value. Ultrasound combined with low doses of chemotherapy drugs can have equally curative effects and fewer side effects. Therefore, LIUS may represent a promising treatment modality for enhancing the local effectiveness of chemotherapeutic drugs.

In the present study, we assessed the proliferation and activity of human OSCC lines (SAS, HSC-3 and HSC-4) under differing ultrasonic intensities and durations at a given frequency. Then, we defined the relationship between cell membrane morphological changes and intracellular stress responses at a LIUS of 1 W/cm^2^ at different incubation times. We also quantitatively assessed the effectiveness of DOX treatment in combination with LIUS by measuring the expression of collagen fibers, MMP-2, MMP-9, and upstream Hh signaling pathway proteins in in vitro and in vivo experiments. Our results provide an experimental basis for better understanding of the mechanism of LIUS in improving DOX efficacy.

## Methods

### Reagents

3-(4,5-Dimethyl-2-thiazolyl)-2,5-diphenyl-2H-tetrazolium bromide (MTT), 2-(4-amidinophenyl)-6-indolecarbamidine dihydrochloride (DAPI) and DOX (D1515) were purchased from Sigma-Aldrich (St Louis, MO, USA). Rabbit anti-LC3 (12135–1-AP) was purchased from Proteintech (Chicago, IL, USA), rabbit anti-MMP-2 (ab37150) and rabbit anti-MMP-9 (ab38898) were purchased from Abcam (Cambridge, MA, USA), rabbit anti-Shh (bs-1544R), rabbit anti-Gli-1 (bs-1206R) and rabbit anti-Beclin 1 (bs-1353R) were purchased from Beijing Bioss Biosynthesis Biotechnology (Beijing, China), and mouse anti-β-actin (sc-47,778) was purchased from Santa Cruz Biotechnology (Santa Cruz, CA, USA).

### Cell lines and culture

Human OSCC cell lines SAS, HSC-4, and HSC-3 and normal HT293 cells were purchased from the Human Science Research Resources Bank (Osaka, Japan) and the Cell Bank of Type Culture Collection of the Chinese Academy of Sciences (Shanghai, China). The cells were cultured in RPMI-1640 supplemented with 10% fetal bovine serum (FBS) and 1% penicillin-streptomycin in a humidified atmosphere containing 5% CO_2_ at 37 °C. Exponentially growing cells were used in the experiments.

### Animals and tumor model

Male, 4-week-old BALB/c nude mice were purchased from SLAC Laboratory Animal Company (Shanghai, China) (*N* = 24). SAS cell suspensions (1 × 10^6^ cells/ml) were subcutaneously injected into the right-side flanks of mice. When the tumor volume reached 100 mm^3^ in size (about 7 days after inoculation), the tumor-bearing mice were randomly divided into four groups. Tumor diameters were measured with calipers and calculated using the following formula: *V* = π/6 × *L* × *S* ^2^, where *L* and *S* are the long and short diameters of the tumors. All animal protocols were approved by the Laboratory Animal Committee of the Harbin Medical University.

### Clinical samples

Archived paraffin-embedded OSCC and matched adjacent normal tissues were obtained from 74 patients who had undergone surgical excision at the Harbin Medical University Stomatological Hospital between January 2006 and December 2011. Patient clinical characteristics have been described previously [7]. All patients gave informed consent, and the study was approved by the Research Ethics Committee of Harbin Medical University (Harbin, China).

### Ultrasonic exposure

The ultrasonic generator and power amplifier used in this study were designed and manufactured by the Harbin Institute of Technology (Harbin, China). The ultrasonic setup is described in our previous publication [24] (Additional file 1: Figure S1). For in vitro experiments, the sound pressure level distribution was simulated by finite element simulation using COMSOL as shown in Additional file 1: Figure S2A & S2B. The ultrasonic transducer (diameter: 40 mm; center frequency: 1.1 MHz; duty factor: 20%; repetition frequency: 100 Hz) was made of a PZT disk attached to a 2.5 cm thick aluminum block serving as the acoustic transmission medium. The LIUS intensities in the culture plate were also measured using a PT0907110 needle-type hydrophone (0.2 cm active element size, 1–10 MHz bandwidth) (Beijing, China). The peak acoustic pressure was 2.6 MPa and the temporal intensity distribution from the center of the transducer along the direction perpendicular to the transducer is shown in Additional file 1: Figure S2a & S2b. For the in vivo experiments, the sound pressure level distribution is shown in Additional file 1: Figure S2C & S2D. The ultrasonic transducer (diameter: 30 mm; center frequency: 1.1 MHz; duty factor: 20%; repetition frequency: 100 Hz) was attached to a tapered aluminum buffer head with a 5 mm diameter front surface that was positioned directly in contact with the skin at the tumor site using an acoustic couplant. The temperature increase in the solution was less than 2 °C in all experiments.

### DOX and ultrasonic treatment of cells in vitro and in vivo

For both in vitro and in vivo experiments, four treatment groups were used: 1) non-treatment (Control); 2) DOX; 3) low intensity ultrasound (LIUS); and 4) DOX in combination with ultrasound (DOX + LIUS).

For the in vitro experiments, cells in the DOX and DOX + LIUS groups were incubated with DOX in the dark. For the control and LIUS groups, an equivalent amount of media was added in place of DOX. After 4 h incubation, the cells in the LIUS and DOX + LIUS groups were exposed to ultrasound (1.1 MHz, 1 W/cm^2^, 20% duty cycle) for 3 min in the dark. After the treatment, the cells were collected for further analyses.

For the in vivo experiments, DOX solution was intraperitoneally injected into tumor-bearing mice in the DOX and DOX + LIUS groups at a dosage of 3 mg/kg, 2 times/week. For comparison, 0.9% normal saline solution was injected into tumor-bearing mice in the control and LIUS groups. Tumors were irradiated by ultrasound (1.1 MHz, 1 W/cm^2^, 20% duty cycle) for 5 min in the dark. The treatment was repeated twice a week for 2 weeks. Tumor diameters and mouse body weights in each group were measured for 14 days. For the prognosis of animals (*N* = 20), the mice were observed daily and sacrificed if they lost 15% of their body weight. The solid tumors removed from different groups were processed for western blotting, immunohistochemistry and Masson’s trichrome staining.

### Cell viability and apoptosis assay

The three human OSCC lines were seeded on detachable 96-well plates and subjected to different treatments for the four groups in each cell line. Then, all groups were cultured for 24 h. Cell viability was quantified by the MTT assay.

In the LIUS and DOX + LIUS groups, the apoptotic cells were detected using Hoechst 33,258 (Sigma-Aldrich) according to the manufacturer’s instructions. Nuclei were visualized by fluorescence microscopy (Olympus, BX51, Japan) with an excitation wavelength of 355 nm and emission wavelength of 465 nm.

### Immunofluorescence

All OSCC cells were fixed in methanol for about 30 min. Then the cells were blocked with 1% BSA for 20 min and incubated with primary antibody (LC3: 1:50; Beclin 1: 1:100) at 4 °C overnight. After rinsing in PBS, the cells were incubated in secondary antibody for 1 h followed by counterstaining with DAPI. The cells were examined by fluorescence microscopy (Olympus, BX5, USA).

### Scanning electron microscopy (SEM)

After exposure to different intensities of LIUS, cells were fixed in 2.5% glutaraldehyde in 0.1 M PBS (pH 7.2–7.4) for 24 h. The cells were then immobilized in 1% osmium tetroxide (OsO_4_), washed with PBS, dehydrated with graded alcohol, displaced, and dried at a critical point. A thin layer of gold was evaporated onto the surface before observation under a scanning electron microscope (S-3400 N, Hitachi, Japan).

### Transmission electron microscopy (TEM)

Cells were fixed in 2.5% glutaraldehyde overnight. After washing with PBS, the samples were dehydrated with graded alcohol and embedded in Epon812 for 72 h at 60 °C. Ultra-thin sections were cut, stained with uranium acetate and lead citrate, and then observed under a transmission electronic microscope (Hitachi, Tokyo, Japan).

### Transwell assays

In vitro migration/invasion activities were assessed using a BD BioCoat™ Matrigel™ Invasion Chamber (BD Biosciences, San Jose, CA, USA). Invasion assays were performed with Falcon cell culture inserts containing 8-μm pore size polyethylene terephthalate membranes with a thin layer of matrigel-reconstituted basement membrane. The cancer cells were seeded into the upper chamber (2 × 10^4^ cells/well). After 24 h incubation at 37 °C, non-invading cells were removed from the upper surfaces of the membranes by scrubbing with cotton-tipped swabs. Invading cells were fixed with methanol, stained with Giemsa and counted under a microscope. In vitro migration assays were performed according to the same procedure but using PET membranes that were not coated with matrigel.

Cell migration and invasion were quantified by counting the number of cells in 10 visual fields on the lower surface of each filter using phase-contrast microscopy.

### Western blotting

Cells and tissues were lysed in RIPA buffer, separated by 10% SDS-PAGE, and transferred to nitrocellulose membranes. After blocking with 5% non-fat dried milk, membranes were incubated with primary antibodies (LC3: 1:500; Beclin 1: 1:500; MMP-2: 1:1000; MMP-9: 1:1000; Shh: 1:100; Gli-1: 1:100) at 4 °C overnight and then incubated with horseradish peroxidase-conjugated secondary antibodies for 1 h. Signals were detected using an enzymatic Chemiluminescence Kit (Pierce, Rochford, IL, USA). β-actin (1:500) was used as an internal control.

### Histologic examination

Analysis and evaluation of immunohistochemical results were performed as previously described [25]. The concentrations of primary antibody were 1:100 for Shh and Gli-1, and 1:500 for MMP-2 and MMP-9.

Masson’s trichrome staining was used to observe deposited collagenous fibers [22, 26]. Collagen fibers stained blue and muscle fibers stained red. The extent of collagenous fiber expression was assessed per unit area.

### Intracellular DOX measurement

The cells of DOX and DOX + LIUS groups were pretreated with 0.2 μg/ml for 3 h prior to sonication. For observation of cellular uptake of DOX using fluorescent microscopy (Olympus, BX5, USA), the cells were fixed in 4% paraformaldehyde for 10 min. The cells were then permeated with 0.1% Triton X-100 for 5 min. For quantification of intracellular DOX concentrations, the cells in 10 randomly selected fields of view (magnification ×200) were counted, and the fluorescence intensity index was used as a measure of cell DOX uptake.

### Statistical analysis

Data are expressed as means ± standard deviations (SD) of three independent experiments. The data were analyzed by one-way ANOVA. Survival analysis was estimated using the Kaplan-Meier method and compared using the log-rank test. The Spearman’s rank correlation coefficient test was used to examine correlations between the expression of collagen fibers, MMP-2, MMP-9, Shh and Gli-1. Results were considered significant when *p < 0.05.*

## Results

### Effects of LIUS and DOX on OSCC cell viability

**Fig. 1:**
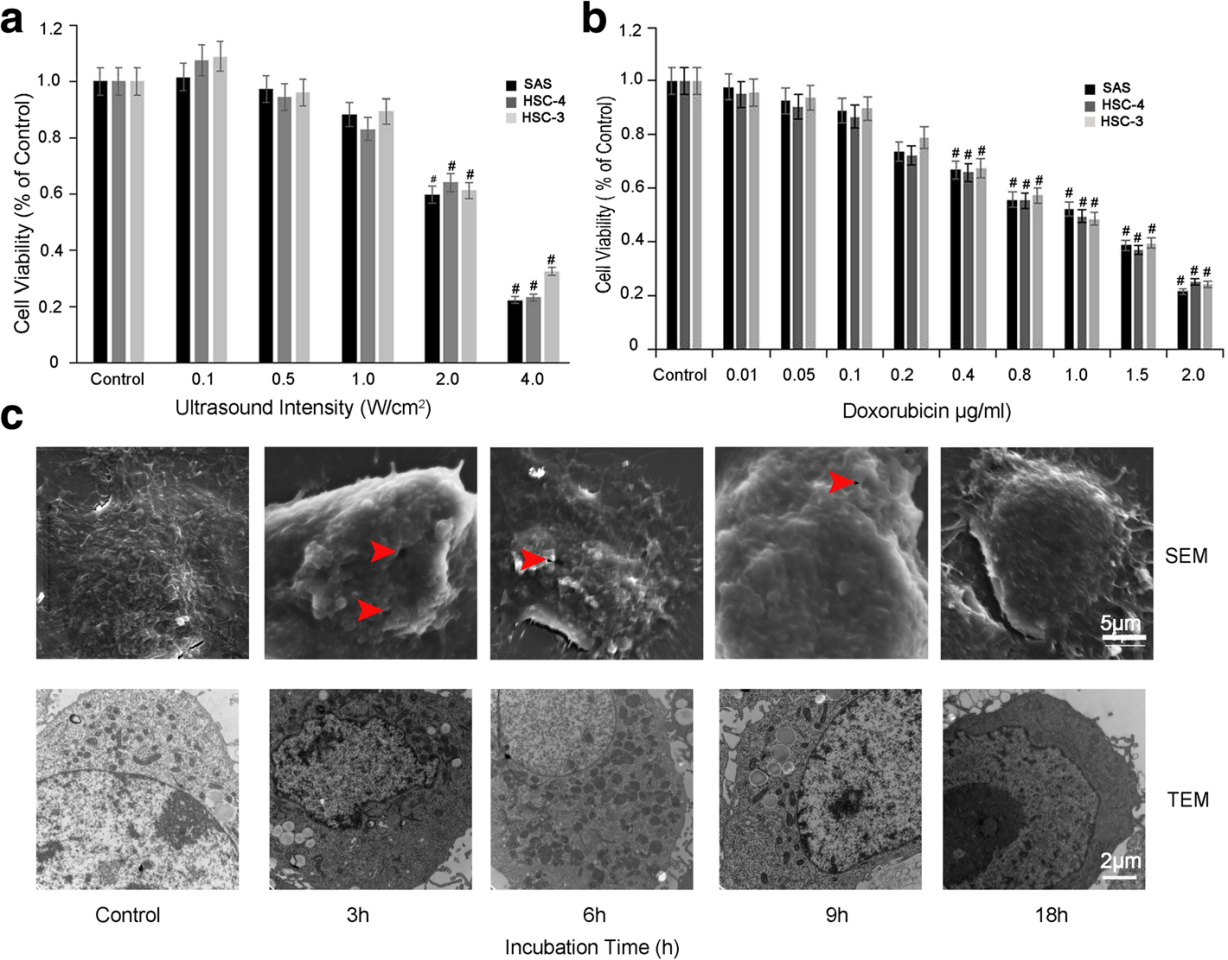
Cytotoxic effects of LIUS and DOX on OSCC cells. Cytotoxic effects were measured by MTT assay after exposure to different ultrasound intensities (**a**) and different DOX dosage (**b**) for 24 h. Data are expressed as means ± SD of three independent experiments. **c,** The morphological and ultra-structural changes of SAS cancer cells were detected by SEM (*upper panel*) and TEM *(lower panel*), at different incubation times after 1 W/cm^2^ ultrasound exposure

To examine the effect of different ultrasonic intensities (0.1, 0.5, 1.0, 2.0, 4.0 W/cm^2^) on OSCC cell viability, we performed sonication followed by MTT assay. The viability rates of the OSCC cells decreased significantly with increasing sonication intensity. The IC50s were 2.332 W/cm^2^ for SAS cells, 2.344 W/cm^2^ for HSC-4 cells and 2.647 W/cm^2^ for HSC-3 cells (Fig. 1a). These results indicate that ultrasound alone can inhibit the growth of OSCC cells in an intensity-dependent manner, though practically no effect was observed for ultrasound intensities below 1.0 W/cm^2^.

To examine the effect of various dosages (0.01, 0.05, 0.1, 0.2, 0.4, 0.8, 1.5, 2.0 μg/ml) of DOX on cell viability, we measured SAS, HSC-4 and HSC-3 cell viabilities by MTT assay after incubation with DOX for 24 h. The cell viabilities decreased significantly with increasing DOX (*p* < 0.01, versus control) (Fig. 1b). The IC50 values for SAS, HSC-4 and HSC-3 were 1.161 μg/ml, 0.811 μg/ml and 0.876 μg/ml, respectively (Fig. 1b). These results suggest that DOX inhibits the growth of OSCC cell lines in a dose-dependent manner, but that there is practically no effect when the DOX dosage is below 0.2 μg/ml.

### Effects of ultrasound on the in vitro cell ultrastructure

Next, we used SEM to detect morphological changes of SAS cells at different incubation times. As shown in Fig. 1c, “pores” appeared on the cell surface after LIUS (1.0 W/cm^2^) treatment for different incubation times. Moreover, the “pores” could be observed up to 9 h after treatment and disappeared completely at 18 h after treatment (Fig. 1c, upper panel). These results indicate that 1.0 W/cm^2^ ultrasound exposure induced transient “pores” on the cell surface, but that the damage was not permanent and the cells self-recovered upon extended incubation.

To further examine the effects of ultrasound on OSCC, we used TEM to study the ultrastructure of SAS cells after 1.0 W/cm^2^ LIUS treatment for different incubation times. As for the SEM, “pores” were visible on the cell membranes, but the membrane structure was still intact, with more cytoplasmic content and clustered nuclear chromatin. At extended incubation times, more cells had autophagic vacuoles containing cell organelles, such as mitochondria and/or endoplasmic reticulum. However, 9 h after the LIUS treatment, the numbers of autophagic vacuoles began to decrease, and almost no autophagic vacuoles could be detected after 18 h (Fig. 1c, lower panel). These findings indicate that pore formation and autophagic vacuoles induced by LIUS at 1.0 W/cm^2^ were recoverable.

**Fig. 2:**
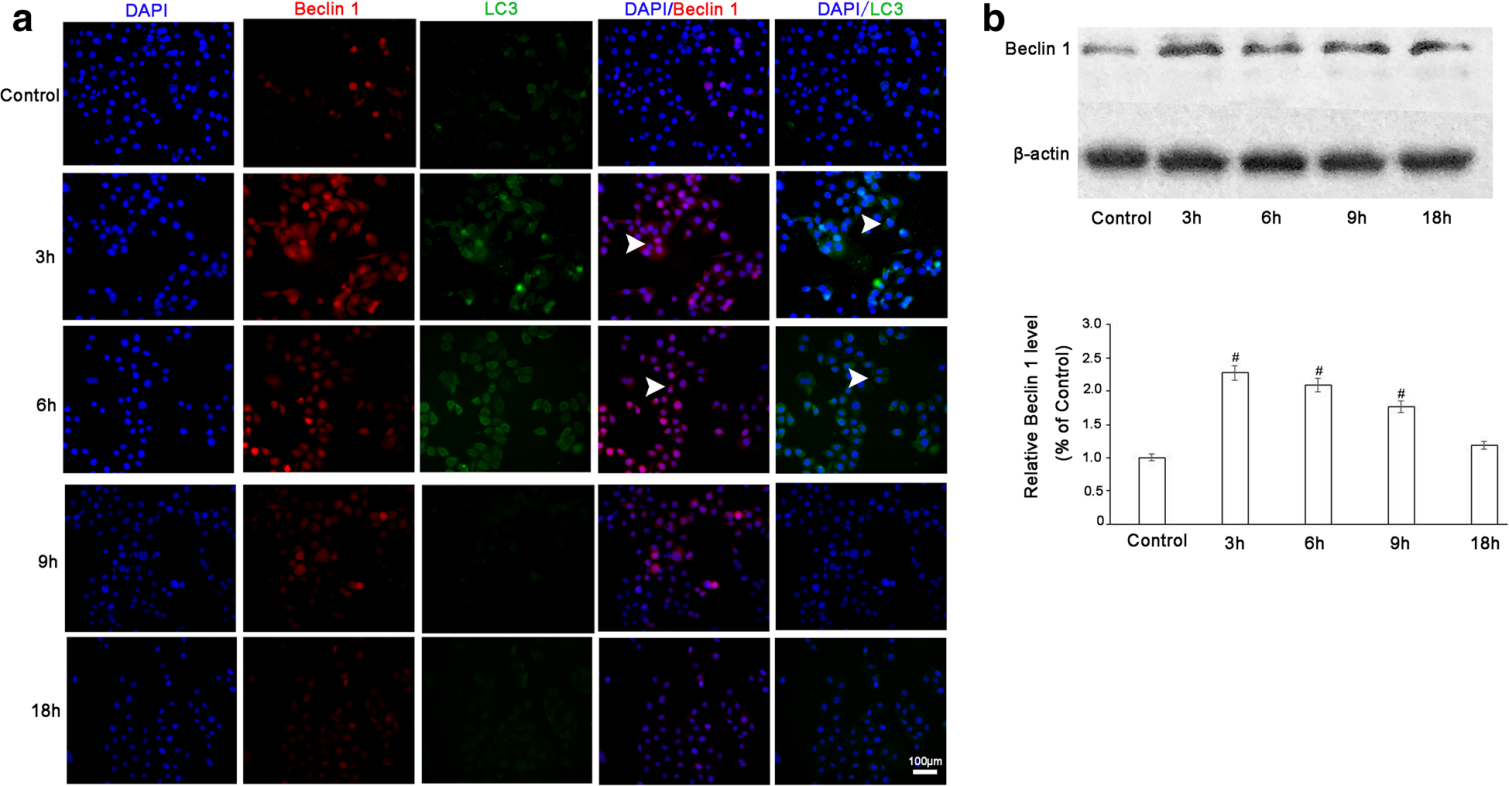
Expression levels of LC3. Immunofluorescence assay (**a**) and Western blotting (**b**) after 1 W/cm^2^ LIUS exposure followed by different incubation times. Data are expressed as means ± SD of three independent experiments. ^**#**^ *p* < 0.05 vs. control group, * *p* < 0.05 vs. 0 h

To further verify these results, we examined the expression of the autophagy markers Beclin1 and LC3 by immunofluorescent microscopy. As shown in Fig. 2a, no expression of Beclin 1 and LC3 was detected in the control group. However, cells treated with LIUS showed increased levels of Beclin 1 and LC3 expression, which subsequently decreased at certain times post-treatment. Western blotting further confirmed the changes in Beclin 1 (Fig. 2b).

### In vitro and in vivo cytotoxicity of combined DOX and LIUS therapy

**Fig. 3:**
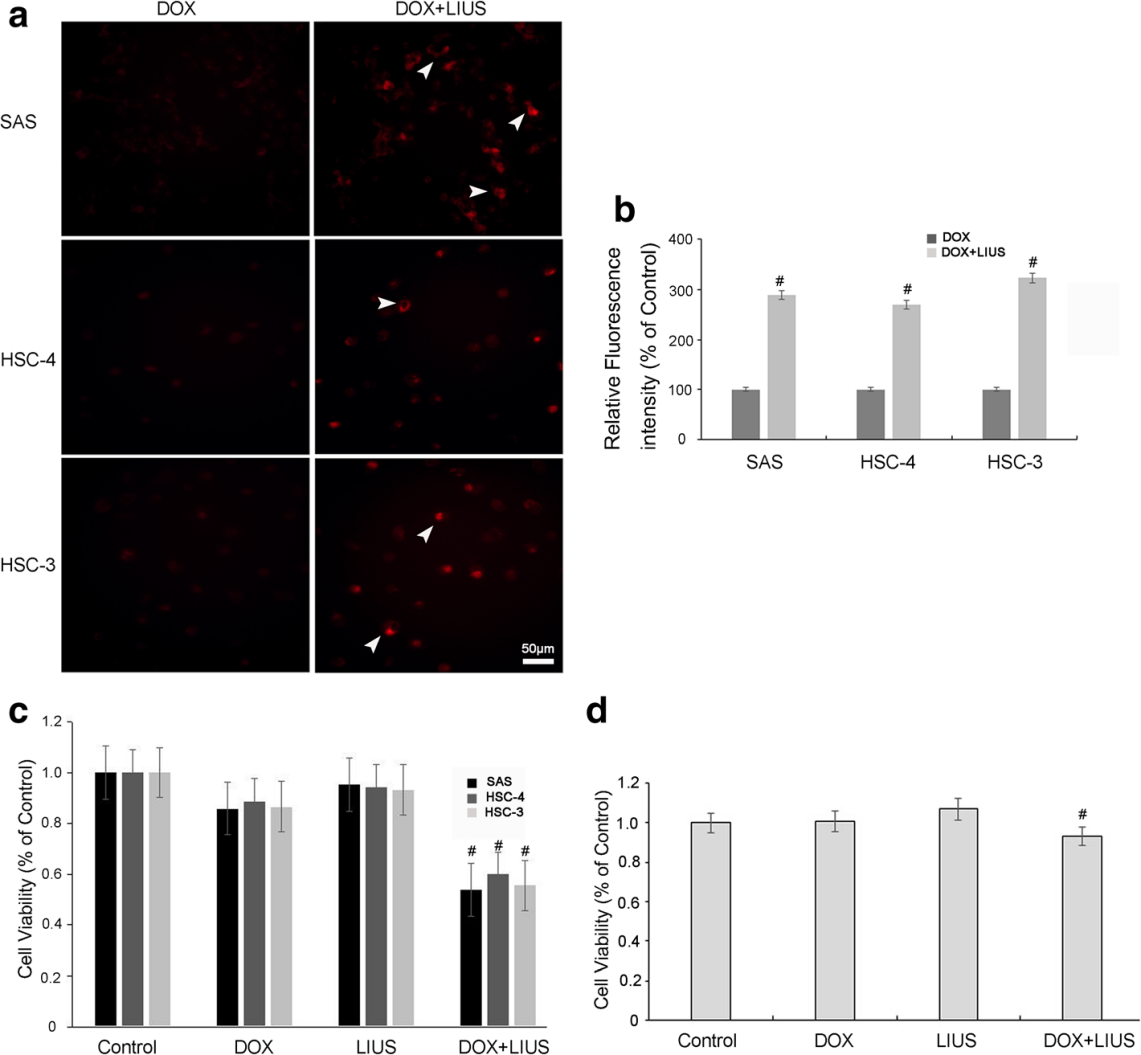
The uptake of DOX and inhibitory effect of DOX and LIUS. **a**, **b**, Fluorescent images of the uptake of DOX in OSCC cells treated with 0.2 μg/ml DOX after LIUS exposure. The cells were treated and incubated for 4 h. Subsequently, the cells were washed and immediately visualized by confocal microscopy. After 24 h, cell viability was evaluated by MTT assay for OSCC cells (**c**) and HT293 cells (**d**)

We further investigated whether “pores” can facilitate the entry of DOX as an extracellular chemotherapeutic drug into cells by examining the intracellular DOX accumulation after LIUS (1.0 W/cm^2^) and low dosage DOX (0.2 μg/ml) combination treatment. As shown in Fig. 3a, DOX was successfully transported into OSCC cells after LIUS treatment (white arrow). Compared to DOX alone, LIUS enhanced DOX uptake by 2.89-fold in SAS cells, 2.69-fold in HSC-4 cells, and 3.23-fold in HSC-3 cells (Fig. 3b). These findings indicate that LIUS exposure dramatically increases the local DOX uptake into OSCC cells.

To examine whether increased DOX entry correlates with enhanced cytotoxic effects, we performed MTT assays 24 h after DOX and LIUS combination treatment. As shown in Fig. 3c, the OSCC cells treated with DOX in combination with LIUS showed significantly decreased cell viability as compared to control cells and cells treated with DOX alone or LIUS alone. Moreover, DOX and LIUS combination treatment did not induce significant cytotoxicity towards non-cancerous HT293 cells (Fig. 3d, and Additional file 1: Figure S3), suggesting that the effects are specific for OSCC cells.

**Fig. 4:**
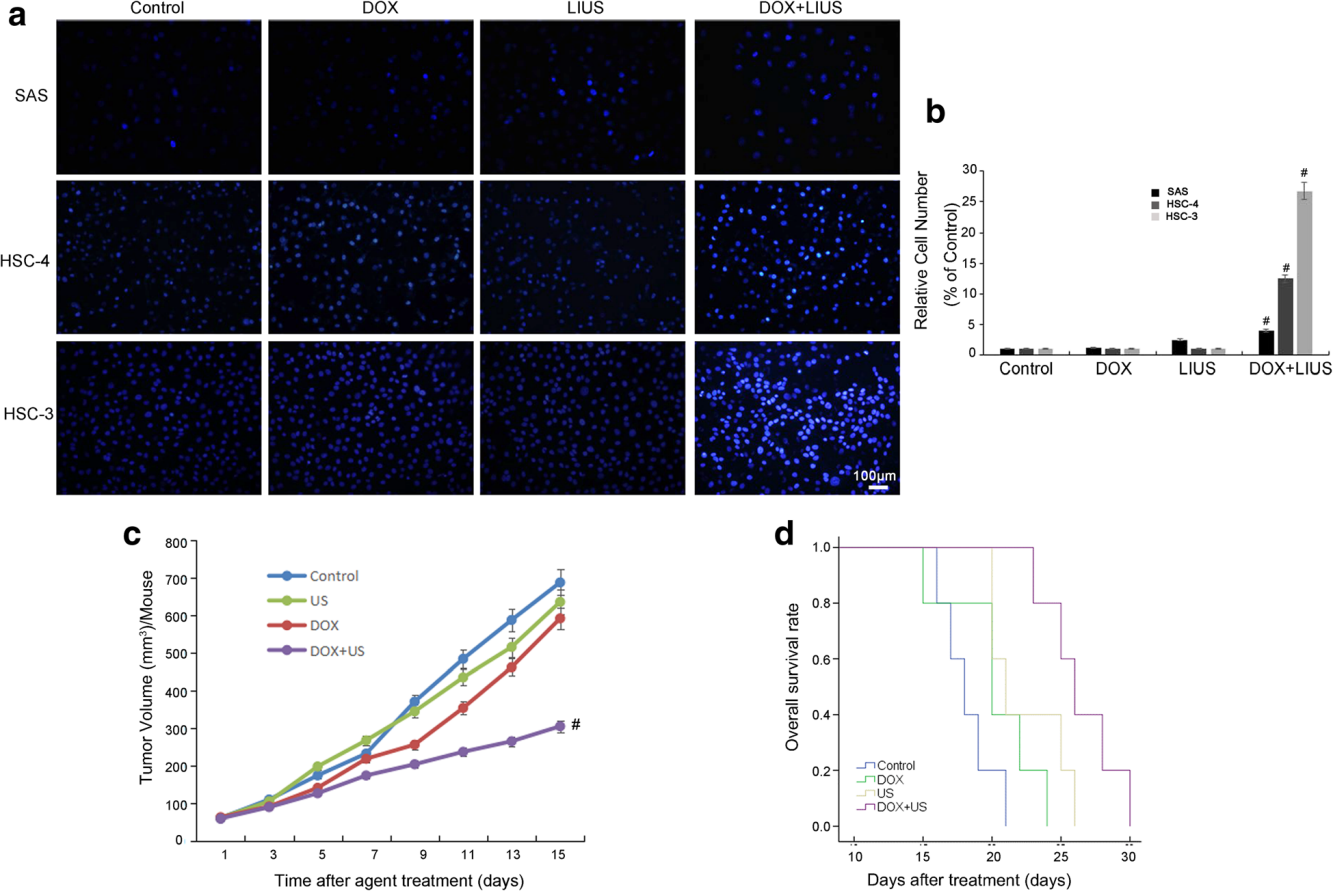
Inhibition of cell proliferation and tumor growth by DOX and LIUS. **a**, **b**, OSCC cells were stained with Hoechst 33,258 at 24 h after treatment, and representative images were obtained using fluorescence microscopy. **c**, DOX in combination with LIUS inhibits SAS tumor growth in BALB/c mice. The tumor volumes per mouse (mm^3^) are presented as means of 5 mouse tumors from each group. **d**, The effect of DOX plus ultrasound on the survival of BALB/c mice transplanted with SAS human OSCC xenografts. The DOX + LIUS group had longer OS compared with Control group (*p* < 0.05). Data represent means ± SD of three independent experiments. ^**#**^ *p* < 0.05 versus each control

As additional verification of the cytotoxic effects of combination treatment of OSCC cells, we examined the nuclei using Hoechst 33,258 staining after combined treatment (Fig. 4a). Compared to the control, DOX alone and LIUS alone groups, the DOX + LIUS group showed an obvious increase in apoptosis for all three cell lines (*P* < 0.05, Fig. 4a and b). These results indicate that DOX in combination with LIUS significantly induces cell apoptosis in cultured OSCC cells.

To evaluate the in vivo biological effects of DOX in combination with LIUS, we employed a SAS tumor xenograft model in nude BALB/c mice. Compared with the control group, mice treated with DOX alone and LIUS alone had no obvious tumor growth suppression, while the DOX + LIUS group showed a significant anti-tumor effect. At the end of the 14-d treatment period, the tumor volume inhibition ratios were 13% in the DOX group, 7% in the LIUS group and 55% in the DOX + LIUS group (Fig. 4c). We also observed a significant increase in the survival rate of mice bearing SAS cells for DOX and LIUS combination treatment compared with DOX alone or LIUS alone treatment (*P* < 0.05, Fig. 4d). Furthermore, no adverse effects, such as skin ulceration or toxic death, were observed in any of the groups.

### Effects of in vitro DOX and LIUS combination treatment on tumor cell migration and invasion

**Fig. 5:**
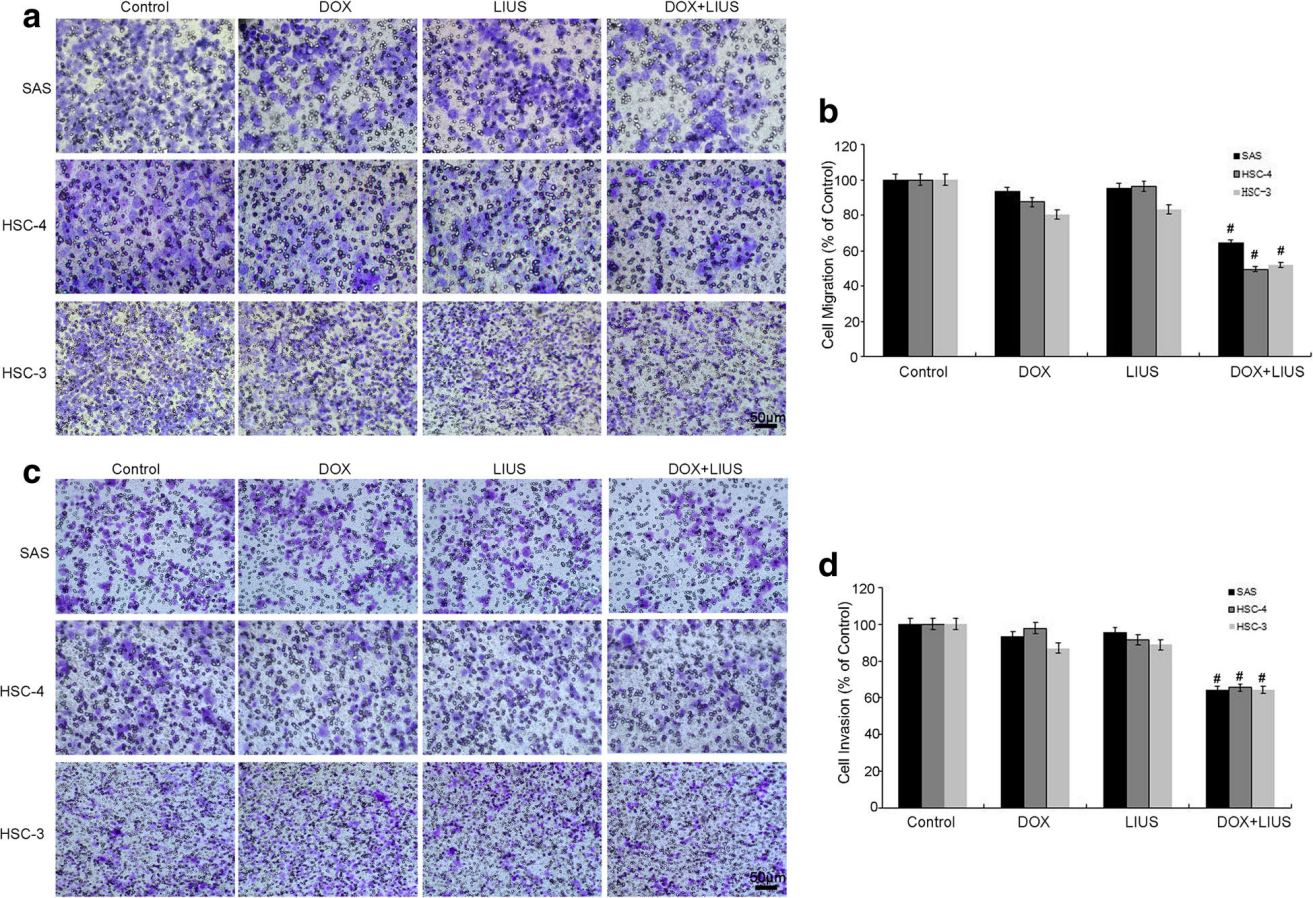
Inhibition of OSCC cell migration and invasion by DOX and in vitro ultrasound. The effect of DOX and LIUS treatment on SAS, HSC-4 and HSC-3 cell migration after 24 h (**a**, **b**) and invasion after 36 h (**c**, **d**) was assessed by Transwell assays. Cells were added into the upper chamber coated with or without matrigel after the indicated treatments. After incubation, the cells that invaded through the matrigel and membrane were stained with crystal violet and quantified. Data are presented as the mean ± SD, *p* < 0.05

We further examined whether DOX in combination with LIUS can inhibit OSCC cell migration and invasion by Transwell assay. Compared to the control group, the LIUS alone and DOX alone groups showed minimal effects on the migration and invasion of OSCC cells. However, the DOX + LIUS group showed significant reduction of cell migration (Fig. 5a and b) and invasion (Fig. 5c and d).

**Fig. 6:**
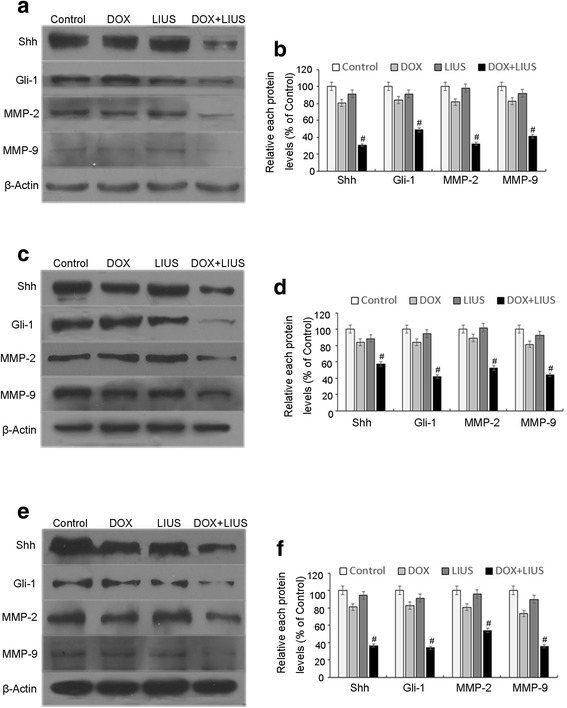
Expression of Shh, Gli-1, MMP-2 and MMP-9. Expression levels of Shh, Gli-1, MMP-2 and MMP-9 proteins were assessed by Western blotting after treatment of SAS cells (**a**, **b**), HSC-4 cells (**c**, **d**) or HSC-3 cells (**e**, **f**). β-Actin was assessed as an internal control. Representative data from three independent experiments are shown. ^**#**^ *p* < 0.05 versus control

To investigate the molecular mechanism of DOX and LIUS combination treatment on cell migration and invasion, we examined effects on signaling molecules that are known to mediate invasiveness, including the Hh signaling pathway mediators Shh and Gli-1 and MMP-2/9. Western blotting assays demonstrated that DOX alone and LIUS alone slightly inhibited the expression of these proteins in OSCC cells. However, DOX in combination with LIUS significantly enhanced inhibition (Fig. 6a and b for SAS cells; Fig. 6c and d for HSC-4 cells; Figs 6e and f for HSC-3 cells). These results are consistent with the possibility that inhibition of tumor migration and invasion by DOX in combination with LIUS may be through the down-regulation of MMP-2, MMP-9, Shh and Gli-1 expression.

### Effects of in vivo DOX and LIUS combination treatment on the expression of collagen, MMP-2/9, Shh and Gli-1

**Fig. 7:**
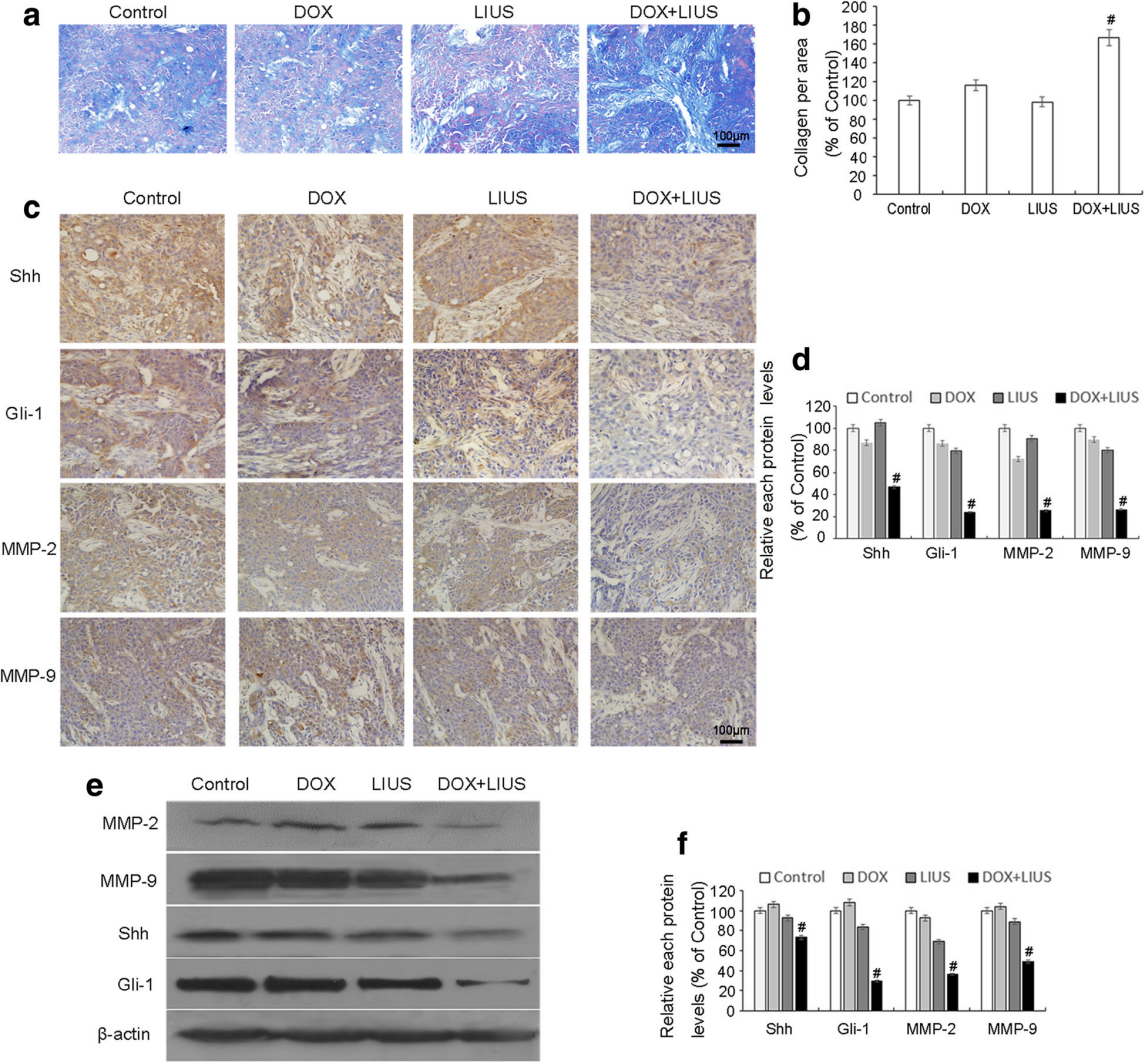
Expression of collagen fibers, MMP-2, 9 and Hh pathway components in BALB/c athymic nude mice. **a**, **b**, Collagen fiber structure in tumors from untreated mice (control) or mice treated with DOX alone, LIUS alone or DOX + LIUS was visualized by Masson’s trichrome staining (^**#**^ *p* < 0.05). The expression of Shh, Gli-1, MMP-2 and MMP-9 in subcutaneous tumors was evaluated by immunohistochemical staining (**c**, **d**, magnification 400×) and Western blot analysis (**e**, **f**). β-actin was assessed as an internal control. Representative data from three independent experiments are shown. Data are presented as the mean ± SD (*n* = 3). * *p* < 0.05 versus control

To determine whether DOX and LIUS combination treatment also reduces the in vivo invasiveness of OSCC, we assessed the effects on SAS tumor xenograft growth in nude BALB/c mice. Our results demonstrate that collagen fibers in the control, DOX alone and LIUS alone groups appeared thin and disrupted, with a lack of fiber cross-linking and a disordered wavy arrangement. However, collagen fibers in the DOX + LIUS group appeared as dense bundles that penetrated into the tumor stroma (Fig. 7a and b). Because collagen fiber is a major component of the BM, these results are consistent with the decreased invasiveness of OSCC cells after treatment with DOX in combination with LIUS .

Next, we assessed the expression of MMP-2, MMP-9, Shh and Gli-1 by immunohistochemical staining (Fig. 7c and d). Expression of all four proteins was significantly down-regulated in the DOX + LIUS group compared with the control group. Western analysis verified these findings (Fig. 7e and f). These results are consistent with the in vitro results suggesting that LIUS may enhance the inhibitory effects of DOX through down-regulation of MMP-2, MMP-9, Shh and Gli-1 expression.

### Expression of MMP-2/9 and Hh signaling pathway components in clinical specimens

**Fig. 8:**
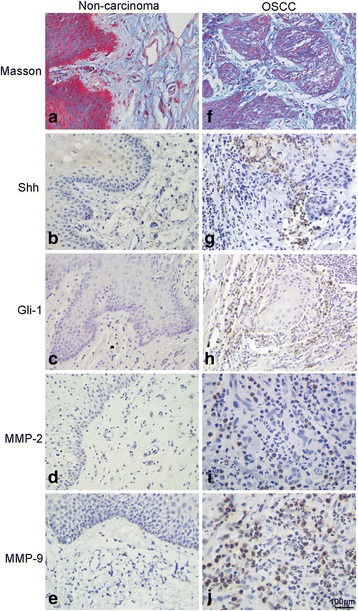
The expression of collagen fibers, MMP-2, 9 and Hh pathway components in malignant OSCC human samples. **a**-**e**, Expression of collagen fibers, MMP-2, 9 and Hh pathway components in non-carcinoma tissues. **f**-**j**, Expression of collagen fibers, MMP-2, 9 and Hh pathway components in human OSCC tissues (200×)

In order to evaluate the potential of this combination treatment strategy for chemotherapy of human OSCC patients, it is important to consider whether the targeted tumor markers are over-expressed in primary human OSCC samples. We evaluated the expression of MMP-2, MMP-9, Shh and Gli-1 in 74 pairs of OSCC tissues and adjacent non-cancerous oral tissue samples. In non-cancerous tissues, collagen fibers were interwoven into a network, and extensive fiber cross-linking was evidenced by thick bundles (Fig. 8a). By contrast, in OSCC tissues, the collagen fibers were thin and short, with reduced cross-linking and a disordered wavy appearance (Fig. 8f). Furthermore, the immunoreactivity of MMP-2, MMP-9, Shh and Gli-1 was increased in OSCC tissues compared with matched adjacent non-cancerous oral tissues (Figs. 8b-e, g-j). We further investigated the correlation between collagen fibers and the expression of MMP-2, MMP-9 and the Hh pathway components in human OSCC samples by Spearman analysis. As shown in Table 1, collagen fiber expression correlated negatively with the expression of each of these proteins. These results suggest that the expression of MMP-2, MMP-9, Shh, and Gli-1 are up-regulated in human OSCC tissues compared with non-cancerous tissues, which provides a rationale for applying DOX and LIUS in combination in future clinical studies.

**Table 1:** Association between expression of collagen fiber and MMP-2, MMP-9, Shh and Gli-1 in patients with OSCC by Spearman’s correlation analysis

Molecule	Collagen fibers	
	Association coefficient (R)	*P* value
MMP-2	−0.271	0.019^#^
MMP-9	−0.459	0.000^#^
Shh	−0.351	0.002^#^
Gli-1	−0.297	0.010^#^

*R* Spearman’s correlation coefficient

^#^ Statistically significant values (*p* < 0.05)

## Discussion

Within a certain range of intensity, the proliferation of cancer cells increases significantly with increases in the sonication intensity. However, ultrasound intensities above a certain threshold cause cellular structural and functional changes. Consequently, biological ultrasound induces cell proliferation or changes in cellular structures or functions [27]. By performing MTT assays, we demonstrated that the viability rates of OSCC cells decreases significantly with increasing sonication intensity (ranging from 0.5–4 W/cm^2^). Compared to the cell viability of the control group, there was no effect on OSCC cell viability when the ultrasound intensity was 1.0 W/cm^2^ (Fig. 1a).

Using SEM, we also found that “pores” immediately formed in cell membranes after 1 W/cm^2^ LIUS exposure, and that they completely disappeared after 18 h incubation (Fig. 1c). Therefore, we speculate that the “pores” are self-recoverable. Intracellular autophagic bodies induced by LIUS were also observed under TEM, which may be indicative of a cellular stress response (Fig. 1c). We demonstrated dynamic expression of the autophagy marker proteins LC3 and Beclin1 after ultrasound treatment, whereas the viabilities of cancer cells were unaffected, possibly because autophagy functions as a protective response to LIUS, as demonstrated in previous studies [21, 28, 29]. We speculate that 1 W/cm^2^ LIUS causes “pores” on the cell surface, and that autophagy that is subsequently activated in response to stress protects the cells from injury [30, 31]. However, the ultrasound intensity used in this study does not produce sonoporation, so whether the “pores” on the cell surface induced by ultrasound rely on cell membrane disruption and/or caveolae-independent endocytosis needs further confirmation.

In order to verify the intracellular DOX concentration after 1 W/cm^2^ LIUS treatment, we assessed the intracellular DOX levels by immunofluorescence. The results showed that after LIUS exposure, the cellular uptake of DOX increased, the activity was significantly inhibited, and apoptosis was increased. This occurred regardless of cell differentiation. Moreover, MTT assays showed no effect for normal HT293 cells treated under the same conditions. Thus, these results indicate that the increased in vitro cytotoxicity of DOX to human OSCC after ultrasound exposure is associated with increased intracellular DOX.

MMP-2 and MMP-9 are known to degrade the BM and ECM, and this degradation facilitates migration and invasion of tumor cells. Some studies suggest that the PI3K/AKT signaling pathway can promote tumor invasion and metastasis by controlling the expression of MMP-2 and MMP-9 [32]. Furthermore, combined LIUS and doxorubicin treatment may inhibit PI3K/Akt/NF-κB pathway activation [33]. Our results suggest LIUS and DOX combination treatment suppresses tumor growth, prolongs survival, increases the expression of MMP-2 and MMP-9, and promotes the deposition of collagen fibers in OSCC xenograft. These results indicate that a relative increase of collagen fibers can affect the invasion capacity of tumors. However, a previous study indicates that sufficient ultrasound intensity can damage interstitial collagen fibers, thereby reducing the compression of tumor vessels and causing increased blood flow and fast transfer of drugs to hypoxic areas, which increases the toxicity of chemotherapy [34]. In our experiments, no destruction of collagen fibers by LIUS was observed due to the use of different ultrasonic parameters or different tumor types.

As an upstream signaling activator of MMP-2 and MMP-9, the Hh signaling pathway plays an important role in the angiogenesis of adult mammalian animals and in cell migration [35, 36]. In consideration of the Shh and Gli-1 results, we can speculate that the decreased MMP-2 and MMP-9 levels may be due to the inhibited activity of the Hh signaling pathway by high intracellular DOX concentrations, thus inhibiting tumor invasion and migration. The relationship between DOX and the Hh signaling pathway is definitely a worthwhile research topic for future investigation.

In addition to increased drug uptake, LIUS can enhance the sensitivity of drug-resistant cells, which provides a treatment strategy for multidrug resistance tumors [37]. Furthermore, DOX is an acoustic-sensitive agent that produces reactive oxygen and free radicals, which can further increase its cytotoxicity [38]. Future evaluation may clarify its mechanisms.

To validate the clinical significance of our results obtained from cell culture and animals, we collected clinical OSCC tissue samples from patients. We observed changes in the amount and morphology of collagen fibers, as well as increased expression of MMP-2, MMP-9, and their upstream regulators Shh and Gli-1, which provides a basis for targeting these signaling molecules. We speculate that this new model of adjuvant chemotherapy will allow the use of greatly reduced DOX doses in order to reduce systemic side effects. Therefore this regimen provides a promising new approach for the treatment of OSCC.

## Conclusion

In summary, our in vitro and in vivo studies confirm that combination treatment with LIUS and DOX leads to the formation of temporary cell surface pores in OSCC cells, which in turn increases the cellular uptake of DOX. Combination treatment also down-regulates Hh signaling and decreases MMP-2 and MMP-9 expression. Furthermore, combination treatment leads to the aggregation of interstitial collagen fibers, which may inhibit tumor invasion to the surrounding tissues, resulting in prolonged survival times for tumor-bearing mice. As a non-invasive, targeted treatment, DOX and LIUS combination treatment overcomes the drawbacks of conventional clinical treatment modalities, therefore providing a useful approach for the treatment of OSCC.

## Additional file

Additional file 1:
**Figure S1.** Schematic diagram of the low-intensity ultrasound device and experimental setup. **Figure S2.** Schematic diagrams of ultrasound pressure level distribution for in vitro and in vivo experiments. **Figure S3.** The effects of LIUS and DOX on HT293 cell. (PPT 734 kb)

## Abbreviations

BM: Basement membrane
DOX: Doxorubicin
ECM: Extracellular matrix
Hh: Hedgehog
IHC: Immunohistochemical
LC3: Light chain 3
LIUS: Low intensity ultrasound
MMP-2: Matrix metalloproteinase-2
MMP-9: Matrix metalloproteinase-9
MMPs: Matrix metalloproteinases
OSCC: Oral squamous cell carcinoma
SEM: Scanning electron microscopy
Shh: Sonic hedgehog
TEM: Transmission electron microscopy
WB: Western blotting
