# Supplementary material for: Doxorubicin combined with low intensity ultrasound suppresses the growth of oral squamous cell carcinoma in culture and in xenografts
Source: J Exp Clin Cancer Res. 2017 Nov 21;36:163. doi: 10.1186/s13046-017-0633-y (PMC5696881; doi:10.1186/s13046-017-0633-y)
Supplement: Additional file 1: — Figure S1. Schematic diagram of the low-intensity ultrasound device and experimental setup. Figure S2. Schematic diagrams of ultrasound pressure level distribution for in vitro and in vivo experiments. Figure S3. The effects of LIUS and DOX on HT293 cell. (PPT 734 kb) [file 13046_2017_633_MOESM1_ESM.ppt]

## Slide 1
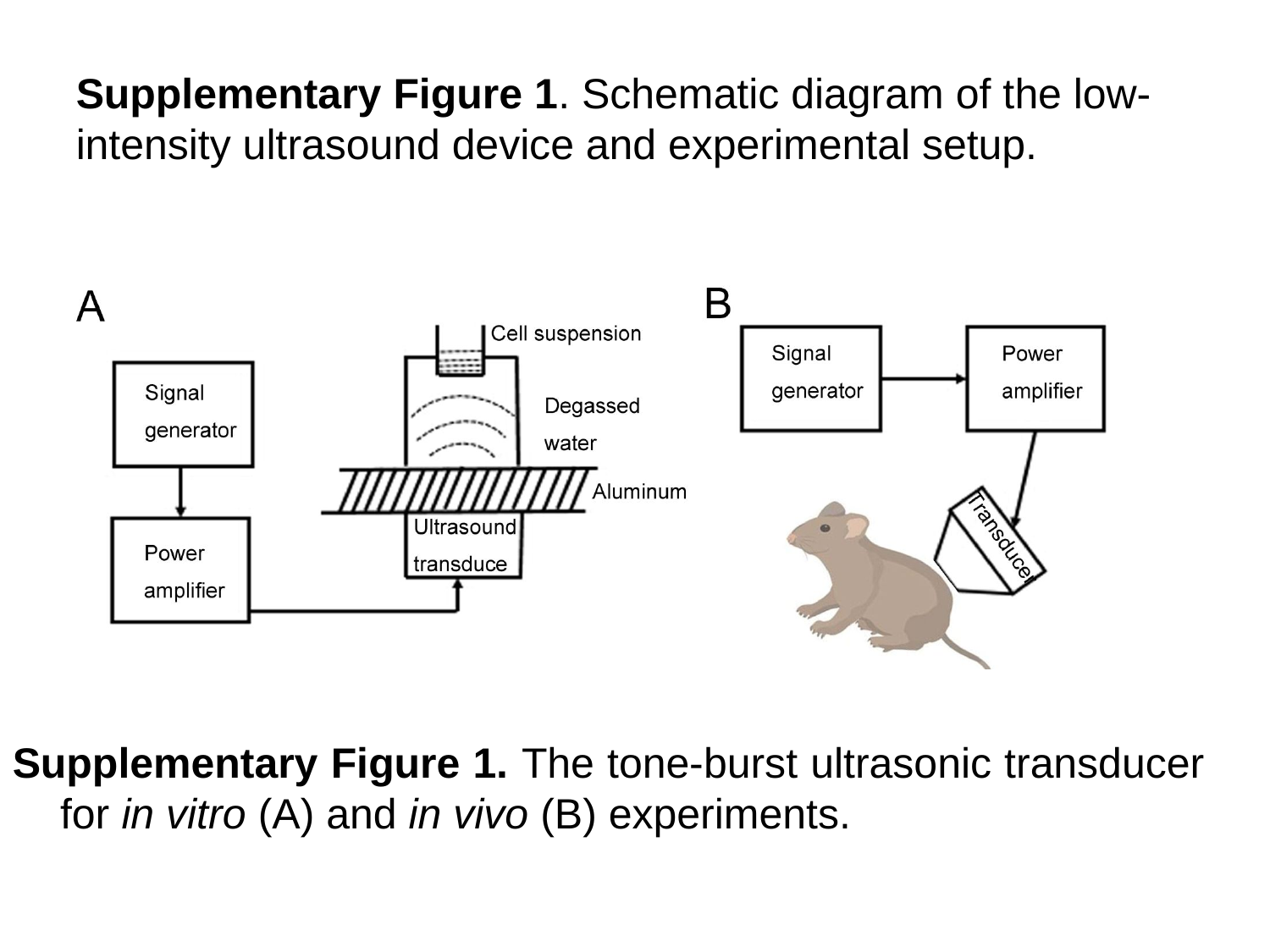

# Supplementary Figure 1. Schematic diagram of the low-intensity ultrasound device and experimental setup.
Supplementary Figure 1. The tone-burst ultrasonic transducer for in vitro (A) and in vivo (B) experiments.

## Slide 2
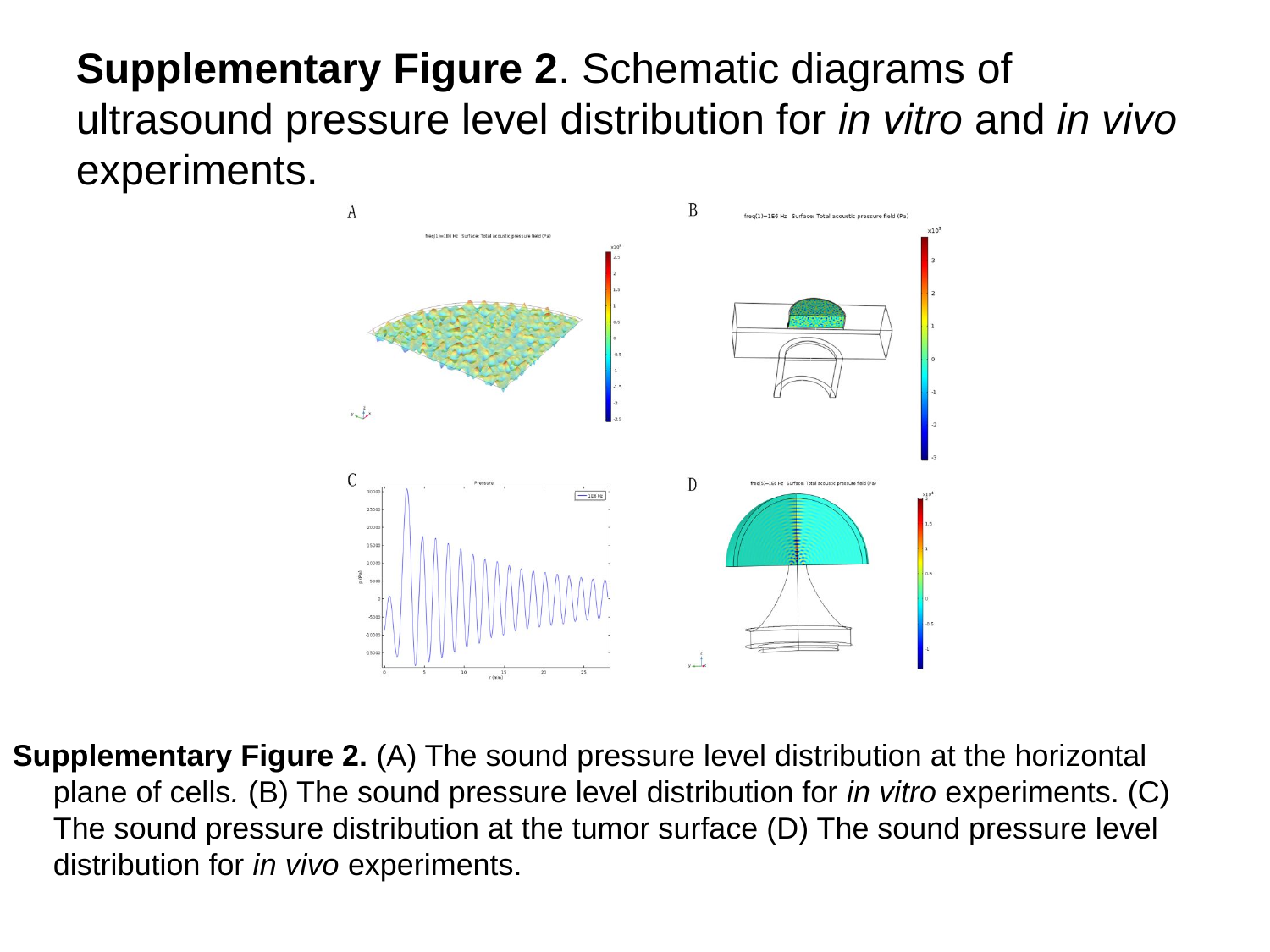

# Supplementary Figure 2. Schematic diagrams of ultrasound pressure level distribution for in vitro and in vivo experiments.
Supplementary Figure 2. (A) The sound pressure level distribution at the horizontal plane of cells. (B) The sound pressure level distribution for in vitro experiments. (C) The sound pressure distribution at the tumor surface (D) The sound pressure level distribution for in vivo experiments.

## Slide 3
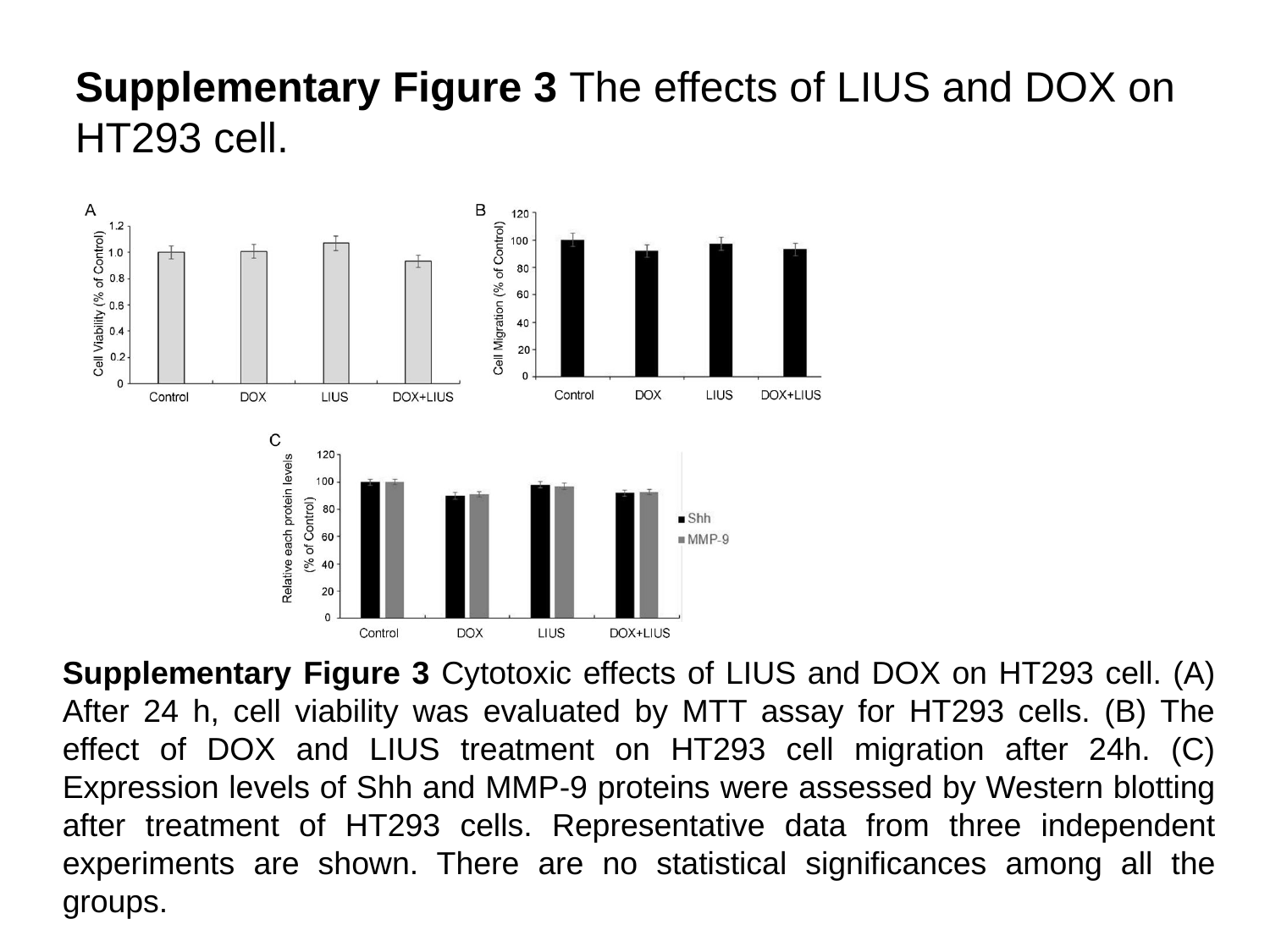

Supplementary Figure 3 The effects of LIUS and DOX on HT293 cell.
Supplementary Figure 3 Cytotoxic effects of LIUS and DOX on HT293 cell. (A) After 24 h, cell viability was evaluated by MTT assay for HT293 cells. (B) The effect of DOX and LIUS treatment on HT293 cell migration after 24h. (C) Expression levels of Shh and MMP-9 proteins were assessed by Western blotting after treatment of HT293 cells. Representative data from three independent experiments are shown. There are no statistical significances among all the groups.
